# ^68^ Ga-FAPI PET/CT versus ^18^ F-FDG PET/CT: Differentiating Metastatic Disease and Reactive Lymph Nodes in a Case of Carcinoma of Breast/Acquired Immunodeficiency Syndrome

**DOI:** 10.1055/s-0044-1787718

**Published:** 2024-06-14

**Authors:** Gopinathraj Gunasekaran, Jaykanth Amalachandran

**Affiliations:** 1 Department of Nuclear Medicine, PET-CT and Theranostics, Apollo Proton Cancer Centre, Chennai, Tamil Nadu, India

**Keywords:** ^68^ Ga-FAPI, reactive node, metastatic node, breast carcinoma, positron emission tomography, PET, acquired immunodeficiency syndrome

## Abstract

Gallium-68 ( ^68^ Ga)-fibroblast activation protein inhibitor (FAPI) positron emission tomography (PET) images the cancer-associated fibroblast that forms a vital component of the tumor microenvironment. It is known that ^68^ Ga-FAPI PET can aid in differentiating reactive lymph nodes from metastatic lymph nodes. ^18^ F-fluorodeoxyglucose (FDG) PET/computed tomography (CT) is still the most commonly used PET radiopharmaceutical in the evaluation of a wide range of malignancies including breast carcinoma. Reactive lymph nodes may also show FDG uptake which can hinder optimal assessment for metastatic involvement. We report an interesting case of invasive ductal carcinoma of the right breast with associated World Health Organization clinical stage I acquired immunodeficiency syndrome for which ^18^ F-FDG PET/CT and ^68^ Ga-FAPI PET/CT were done.

## Introduction

^18^ F-fluorodeoxyglucose (FDG) positron emission tomography/computed tomography (PET/CT) has become the standard in metastatic evaluation of newly diagnosed breast carcinoma. However, it carries a disadvantage in that it cannot differentiate inflammatory tissue from that of metastatic disease. Cancer-associated fibroblasts are an important part of the tumor microenvironment that promote the cancer tissue invasion and metastatic spread in addition to stimulating the angiogenesis and cell growth. These fibroblasts constitute about more than 90% of the tumor mass in most of the epithelial carcinomas such as colon, pancreas, and breast cancer. These cancer-associated fibroblasts express a surface protein called the fibroblast-activated protein (FAP). FAP is a transmembrane glycoprotein with dipeptidyl peptidase-4 and endopeptidase activity. A novel PET imaging agent designed to target and bind with this FAP is FAP inhibitor usually labeled with gallium-68 ( ^68^ Ga). There have been several case reports that reveals the potential role of ^68^ Ga-fibroblast activation protein inhibitor (FAPI) PET in differentiating reactive lymph nodes from metastatic lymph nodes. 1 2 3 Several studies have been done to evaluate the role of ^68^ Ga-FAPI PET/CT in breast carcinoma patients, most of which shows better sensitivity with FAPI PET/CT than the FDG PET. 4 5 6 The pathophysiology behind acute and chronic inflammation can explain the lack of avidity for ^68^ Ga-FAPI in acute reactive lymph nodes. In acute inflammation, neutrophils and lymphocytes infiltrate predominant lymph nodes and play an active role in inflammation mediation, resulting in ^18^ F-FDG uptake. Fibroblast activation regulates the switch from acute inflammation to chronic inflammation resulting in tissue remodeling, fibrosis, and scarring as a result of multiple tissue insults, which may explain the ^68^ Ga-FAPI uptake in chronic inflammation. 7 8

## Case Report

FAP inhibitor is a ligand that targets the FAP receptor expressed in the cancer-associated fibroblasts which are a part of the tumor microenvironment. ^68^ Ga-FAPI PET has become popular because of its ease of availability (in-house production), better target to background ratio, and easier patient preparation compared with the ^18^ F-FDG.

**Fig. 1:**
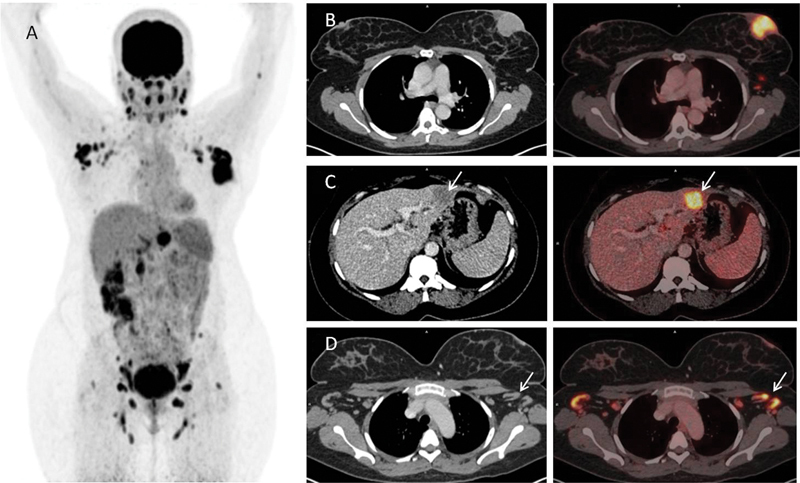
18F-FDG PET/CT : Maximum intensity projection, Axial CT and axial fused PET/CT images (From Left to Right).

Fig. 1 shows a 40-year-old woman with acquired immunodeficiency syndrome and pathologically proven invasive ductal carcinoma of the left breast was referred for metastatic evaluation using ^18^ F-FDG PET/CT. Maximum intensity projection (MIP) image is shown in Fig. 1A . ^18^ F-FDG PET/CT images revealed intensely FDG avid well-defined heterogeneously enhancing mass lesion (4.0 × 3.9 cm, maximum standardized uptake value [SUVmax] 10.4) in the retroareolar region of the left breast infiltrating the nipple areolar complex, multiple enlarged lymph nodes (bilateral cervical I–V, axillary I–III, internal mammary, mediastinal, abdominopelvic, and inguinal lymph nodes), and FDG avid hypoenhancing nodular lesion in the segment II/III of the liver (3.2 × 2.8 cm, SUVmax 10.5) ( Fig. 1B , D , and C , respectively). In such a scenario, it is impractical to differentiate reactive lymphadenopathy secondary to acquired immunodeficiency syndrome from that of the metastatic lymph nodes.

**Fig. 2:**
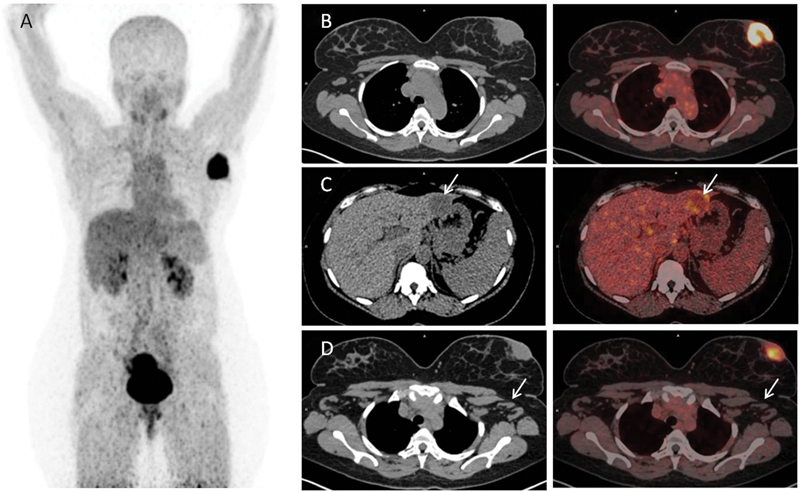
68Ga-FAPI PET/CT : Maximum intensity projection, Axial CT and axial fused PET/CT images (From Left to Right).

Subsequently, ^68^ Ga-FAPI PET/CT was done the next day ( Fig. 2 ). MIP image is shown in Fig. 2A . The primary lesion in the left breast was showing intense FAPI avidity (SUVmax 19.5) ( Fig. 2B ). None of the left axillary lymph nodes were FAPI avid ( Fig. 2D ). However, the liver lesion in segment II/III of the liver showed peripherally increased ^68^ Ga-FAPI uptake (SUVmax 11.5) ( Fig. 2C ). Fine-needle aspiration cytology of bilateral enlarged axillary lymph nodes was done which revealed reactive lymphoid infiltrates. Further, core needle biopsy of segment II/III liver lesion was done under ultrasound guidance, histopathological examination report of which read as poorly differentiated neoplasm favoring metastatic deposit.

Comparing the ^68^ Ga-FAPI PET and the ^18^ F-FDG PET images, FAPI PET is more sensitive in detection of the primary breast lesion and ruling out the acute reactive lymph nodes. However, the liver lesion shows low ^68^ Ga-FAPI uptake. This may be attributed to the tumor heterogeneity representing the presence of tumor-induced fibroblast in the periphery of the lesion and vice versa for ^18^ F-FDG PET.

## Conclusion

18F-FDG PET/CT is considered a standard imaging modality for metastatic evaluation in breast carcinoma. It is imperative to differentiate acute inflammatory reaction from that of metastatic disease for appropriate management which can be difficult with 18F-FDG PET/CT.

^68^ Ga-FAPI PET/CT can be a potential tool in differentiating acute inflammatory pathology from metastatic disease in selective case scenarios.
